# Supplementary material for: Whole genome sequencing analysis of body mass index identifies novel African ancestry-specific risk allele
Source: Nat Commun. 2025 Apr 11;16:3470. doi: 10.1038/s41467-025-58420-2 (PMC11992084; doi:10.1038/s41467-025-58420-2)
Supplement: Supplementary file 2 — Description of Additional Supplementary Files [file 41467_2025_58420_MOESM2_ESM.pdf]

Description of Additional Supplementary Files

Supplementary Data 1	Participant counts by study and population group
Supplementary Data 2	BMI and percent female by study
Supplementary Data 3	BMI and percent female by population group
Supplementary Data 4	Study-specific descriptive statistics of age and BMI
Supplementary Data 5	Genome-wide significant variants by locus and ALT frequency by population group
Supplementary Data 6	Genome-wide significant variants by locus from African and European population group-specific analyses
Supplementary Data 7	Summary of top loci from sensitivity analysis using African subset-specific PCs
Supplementary Data 8	Replication of rs111490516 and rs73396827
Supplementary Data 9	Variant annotation from Variant Effect Predictor (VEP) and FORGEdb summary information for all SNPs in high LD (R2 > 0.8) with top SNP in novel *MTMR3* locus and likely causal SNPs from PAINTOR fine-mapping analysis (PP > 0.5)
Supplementary Data 10	Summary of per locus association results after conditioning on top index variant
Supplementary Data 11	Summary of association results after conditioning on all known index variants
Supplementary Data 12	Rare variant aggregate association analysis for the exome-wide significant genes reported in Akbari et al. (PMID: 34210852)
Supplementary Data 13	PAINTOR results for top loci assuming one single causal variant at each locus
Supplementary Data 14	Top associations meeting suggestive significance (P < 0.001) in PheWAS meta-analysis for rs111490516
