# Supplementary material for: Whole genome sequencing analysis of body mass index identifies novel African ancestry-specific risk allele
Source: Nat Commun. 2025 Apr 11;16:3470. doi: 10.1038/s41467-025-58420-2 (PMC11992084; doi:10.1038/s41467-025-58420-2)
Supplement: Supplementary file 4 — Reporting Summary [file 41467_2025_58420_MOESM4_ESM.pdf]

nature portfolio

Corresponding author(s): Xinruo Zhang and Anne E. Justice

Last updated by author(s): Feb 7, 2025

Reporting Summary

Nature Portfolio wishes to improve the reproducibility of the work that we publish. This form provides structure for consistency and transparency in reporting. For further information on Nature Portfolio policies, see our Editorial Policies and the Editorial Policy Checklist.

Statistics

For all statistical analyses, confirm that the following items are present in the figure legend, table legend, main text, or Methods section.

n/a	Confirmed	
☐	☒	The exact sample size (*n*) for each experimental group/condition, given as a discrete number and unit of measurement
☒	☐	A statement on whether measurements were taken from distinct samples or whether the same sample was measured repeatedly
☐	☒	The statistical test(s) used AND whether they are one- or two-sided<br>*Only common tests should be described solely by name; describe more complex techniques in the Methods section.*
☐	☒	A description of all covariates tested
☐	☒	A description of any assumptions or corrections, such as tests of normality and adjustment for multiple comparisons
☐	☒	A full description of the statistical parameters including central tendency (e.g. means) or other basic estimates (e.g. regression coefficient) AND variation (e.g. standard deviation) or associated estimates of uncertainty (e.g. confidence intervals)
☐	☒	For null hypothesis testing, the test statistic (e.g. *F*, *t*, *r*) with confidence intervals, effect sizes, degrees of freedom and *P* value noted<br>*Give P values as exact values whenever suitable.*
☒	☐	For Bayesian analysis, information on the choice of priors and Markov chain Monte Carlo settings
☒	☐	For hierarchical and complex designs, identification of the appropriate level for tests and full reporting of outcomes
☐	☒	Estimates of effect sizes (e.g. Cohen's *d*, Pearson's *r*), indicating how they were calculated

Our web collection on statistics for biologists contains articles on many of the points above.

Software and code

Policy information about availability of computer code

Data collection	No special software was used to collect these data.
Data analysis	All protocols used for variant calling and quality control for data used in this study are described here: https://topmed.nhlbi.nih.gov/topmed-whole-genome-sequencing-methods-freeze-8. Links to relevant code, including github repositories, are also provided on the same site.<br>All GWAS analyses were performed using GENESIS, a bioconductor package, on the TOPMed Analysis Commons. Details of the GENESIS app used on the Analysis Commons, along with underlying package information and code are provided here: https://github.com/AnalysisCommons/genesis_wdl. All scripts used for running analyses on the TOPMed Analysis Commons on the DNAnexus Platform are provided here: https://github.com/Justice-Genetics-Lab/TOPMed-WGS-BMI-GWAS/tree/main and DOI: 10.5281/zenodo.14708351.

For manuscripts utilizing custom algorithms or software that are central to the research but not yet described in published literature, software must be made available to editors and reviewers. We strongly encourage code deposition in a community repository (e.g. GitHub). See the Nature Portfolio guidelines for submitting code & software for further information.

Data

Policy information about availability of data

All manuscripts must include a data availability statement. This statement should provide the following information, where applicable:

- Accession codes, unique identifiers, or web links for publicly available datasets
- A description of any restrictions on data availability
- For clinical datasets or third party data, please ensure that the statement adheres to our policy

The GWAS summary data generated in this study, including pooled, African, European, and sensitivity, have been deposited in the NHGRI-EBI Catalog of human genome-wide association studies (GWAS Catalog) database under accession codes GCST90502911 to GCST90502914 (https://www.ebi.ac.uk/gwas/downloads/summary-statistics). The raw TOPMed Program individual-level data are protected due to data privacy laws, but de-identified versions are available through Google and AWS cloud services following NIH dbGap approval. Details on gaining accessing are found on the TOPMed website (see https://topmed.nhlbi.nih.gov/topmed-data-access-scientific-community and https://topmed.nhlbi.nih.gov/topmed-whole-genome-sequencing-methods-freeze-8#access-to-sequence-data). In addition to raw data and full GWAS summary statistics provided through the referenced repositories, the summary statistics on the study population used in this study along with summary results for top findings are provided in the Supplementary Data files.

Research involving human participants, their data, or biological material

Policy information about studies with human participants or human data. See also policy information about sex, gender (identity/presentation), and sexual orientation and race, ethnicity and racism.

Reporting on sex and gender	Sex was used as a biological variable in this study. Biological sex was used for the cleaning and quality control (i.e. identifying potential sample swaps), coding of the genetic data (for chr X), and as a covariate in association analyses.
Reporting on race, ethnicity, or other socially relevant groupings	Our study population was racially, ethnically, geographically, and ancestrally diverse. We analyzed a multi-population sample of 88,873 adults from 36 studies in the freeze 8 TOPMed and CCDG programs. They belonged to 15 population groups, reflecting the way participants self-identified in each study. For individuals who had unreported or non-specific population memberships (e.g., "Multiple" or "Other"), we applied the Harmonized Ancestry and Race/Ethnicity (HARE) method to infer their group memberships using genetic data, assigning each to one of the existing population groups based on the group with the highest probability of membership. All other participants remained in the population group assigned based on their self-reported race/ethnicity/population group. In this way, our study population groups were defined based on a combination of self-reported identity and inferred genetic similarity.
Population characteristics	Our study population included only adult participants over the age of 18 with whole genome sequencing data available. Detailed descriptive statistics on our sample are provided by study, population group, and sex (Supplementary Data 4).
Recruitment	This study combined data available from 36 studies with existing genetic and phenotype data; thus, recruitment occurred in a number of different ways (i.e. family-based studies, clinic-based studies, population-based studies).
Ethics oversight	Each participating study obtained IRB approval from the original host institution. The current paper was deemed non-human subjects research as it involved secondary analysis of existing data using de-identified data resources.

Note that full information on the approval of the study protocol must also be provided in the manuscript.

Field-specific reporting

Please select the one below that is the best fit for your research. If you are not sure, read the appropriate sections before making your selection.

☒ Life sciences ☐ Behavioural & social sciences ☐ Ecological, evolutionary & environmental sciences

For a reference copy of the document with all sections, see nature.com/documents/nr-reporting-summary-flat.pdf

Life sciences study design

All studies must disclose on these points even when the disclosure is negative.

Sample size	Sample size was not predetermined. This study was conducted on an opportunistic sample. All subjects with the available phenotype, genotype, and covariate data were included in the analyses.
Data exclusions	Participants were excluded from analyses if genotype data did not meet quality controls standards, if participant was less than 18 years of age, had known pregnancy at the time of BMI measurement, had implausible BMI values (above 100 kg/m2 without corroborating evidence), or did not provide appropriate consent.
Replication	For the novel single-variant association identified in the MTMR3 locus from our discovery analyses, we requested replication specifically in participants from six independent cohorts. We were able to replicate both genetic variants in the novel locus that were requested.
Randomization	This is not relevant to our study as the main trait of interest is a quantitative trait.
Blinding	This is not applicable to this study as we conducted a genetic association analysis and no trial was conducted.

Reporting for specific materials, systems and methods

We require information from authors about some types of materials, experimental systems and methods used in many studies. Here, indicate whether each material, system or method listed is relevant to your study. If you are not sure if a list item applies to your research, read the appropriate section before selecting a response.

Materials & experimental systems

n/a	Involved in the study
☒	☐ Antibodies
☒	☐ Eukaryotic cell lines
☒	☐ Palaeontology and archaeology
☒	☐ Animals and other organisms
☒	☐ Clinical data
☒	☐ Dual use research of concern
☒	☐ Plants

Methods

n/a	Involved in the study
☒	☐ ChIP-seq
☒	☐ Flow cytometry
☒	☐ MRI-based neuroimaging

Plants

Seed stocks	N/A
Novel plant genotypes	N/A
Authentication	N/A
